# Determinants of anemia among 6–59 months aged children in Bangladesh: evidence from nationally representative data

**DOI:** 10.1186/s12887-015-0536-z

**Published:** 2016-01-11

**Authors:** Jahidur Rahman Khan, Nabil Awan, Farjana Misu

**Affiliations:** Centre for Nutrition and Food Security, International Centre for Diarrhoeal Disease Research, Bangladesh (icddr, b), Dhaka, Bangladesh; Institute of Statistical Research and Training, University of Dhaka, Dhaka, Bangladesh; Department of Agricultural Statistics, Bangladesh Agricultural University, Mymensingh, Bangladesh

**Keywords:** Anemia, 6–59 months, Children, Determinants, Bangladesh

## Abstract

**Background:**

Anemia is a global public health problem but the burden of anemia is disproportionately borne among children in developing countries. Anemia in early stages of life has serious consequences on the growth and development of the children. We examine the prevalence of anemia, possible association between anemia and different socio-economic, demographic, health and other factors among children with ages from 6 to 59 months from the nationally representative 2011 Bangladesh Demographic and Health Survey (BDHS).

**Methods:**

Data on hemoglobin (Hb) concentration among the children aged 6–59 months from the most recent BDHS (2011) were used. This nationally representative survey allowed a multistage stratified cluster sampling design and provided data on a wide range of indicators such as fertility, mortality, women and child health, nutrition and other background characteristics. Anemia status was determined using hemoglobin level (<11.0 g/dl), and weighted prevalence of childhood anemia along with 95 % confidence intervals were provided. We also examined the distribution of weighted anemia prevalence across different groups and performed logistic regression to assess the association of anemia with different factors.

**Results:**

A total of 2171 children aged 6–59 months were identified for this analysis, with weighted prevalence of anemia being 51.9 % overall- 47.4 % in urban and 53.1 % in rural regions. Results of a multivariable logistic regression analysis showed that, children below 24 months of age (odds ratio, [OR] 3.01; 95 % confidence interval [CI] 2.38-3.81), and those from an anemic mother (OR 1.80; 95 % CI 1.49-2.18) were at higher risk of anemia. Childhood anemia was significantly associated with chronic malnutrition of child, source of drinking water, household wealth and geographical location (defined by division).

**Conclusions:**

A high prevalence of anemia among 6–59 months aged children was observed in Bangladesh. Given the negative impact of anemia on the development of children in future, there is an urgent need for effective and efficient remedial public health interventions.

## Background

Anemia is a prevalent public health problem which affects about a quarter of the world population [1], notably pre-school aged (PreSAC) children with global prevalence in the 0–5 year-old age group rising to 47.4 % [2]. According to World Health Organization (WHO) criteria, anemia ranks as a severe public health problem (defined as a prevalence of ≥ 40 %). Anemia can adversely affect cognitive advancement, performance in school, physical and behavioral growth, and immunization ability of children against disease [3–6]. It remains a major cause of mortality and morbidity in developing countries where resources to determine the underlying etiology remain poor [3]. According to WHO, Africa has the highest anemia prevalence overall for PreSAC, non-pregnant and pregnant women, where the Asian region shows the highest number of people being affected with 58 % of the anemia burden exists for PreSAC [2]. According to recent information from the South Asian region, the prevalence of anemia among children 6–35 months aged was about 79 % in India. In Nepal, the prevalence among children <5 years was 46 %. The national overall prevalence of Anemia in Bangladesh was approximately 51 % in 2011 [7].

Anemia in children is of particular interest since it can negatively and irreversibly impact their future development. Although the etiology of anemia among children is multi-factorial, the most significant correlates to the onset of childhood anemia is iron deficiency with a smaller proportion due to deficiencies of such micronutrients as folate, Vitamin A and B12 [8–10]. Prevalence of iron deficiency anemia in developing countries varies; Villalpando notes it is frequently four times higher than in developed countries [8].

Several surveys in the past have shown that anemia is a severe problem in Bangladesh among children. In Bangladesh, prevalence of anemia varied across the different surveys which were focused on slightly different populations. According to the Nutritional Surveillance Project (NSP), prevalence of anemia was 47 % in 2001 and 68 % in 2004 among 6–59 months aged children [11]. Anemia tends to reduce with age, and another study notes 64 % prevalence in children aged 6–23 months, and 42 % in children aged 24–59 months in Bangladesh [12]. On the other hand, National Micronutrient Survey in 2011–12 showed an anemia prevalence of only 33 % among 6–59 months aged children, although methodology was different than in other studies [13]. The prevalence of anemia is higher among younger children because their nutritional requirements for growth are high. The underlying causes of anemia among children are multi-factorial and there is no study which works with national level data on anemia and associated factors.

In this study, we performed a comprehensive investigation of childhood anemia and its determinants among the PreSAC children in Bangladesh. Our aim is to estimate the national prevalence of anemia and explore the factors associated with anemia as a basis for prevention and control programs. Moreover, the study can help public health policymakers determine priorities for intervention.

## Methods

**Fig. 1:**
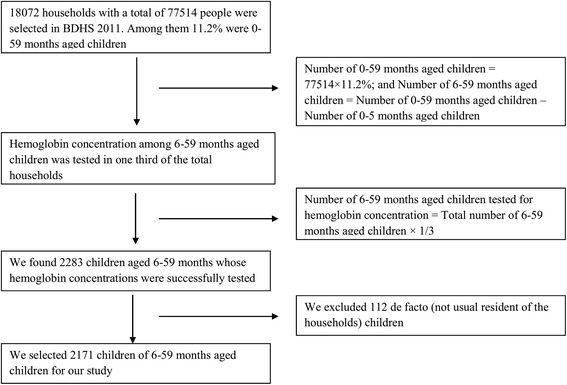
Data selection flow chart

Data on 7481 children with ages from 6 to 59 months born in the last 5 years were extracted from 2011 Bangladesh Demographic and Health Survey (BDHS). This national level survey was designed to provide data on basic indicators of fertility regulation, maternal health, child health, nutritional status of mothers and children, awareness and attitude towards HIV/AIDS, and the prevalence of non-communicable diseases. Enumeration areas (EAs) from the population census 2011 were primary sampling units (PSUs) for this survey, with PSUs designed to produce separate estimates of key indicators for each of the seven divisions such as Dhaka, Chittagong, Rajshahi, Rangpur, Khulna, Barisal and Sylhet. Data collection took place over a five month period from July 8 to December 27, 2011. By using the stratified, two-stage cluster design, where, a total of 600 clusters (including 207 clusters in urban areas and 393 clusters in rural areas) were chosen in first stage [14]. In the second stage of sampling, a systematic sample of 30 households (HHs) was selected on average per cluster. Detailed information about the survey can be found in the 2011 BDHS report [14]. Hemoglobin testing was carried out among children aged 6–59 months in every third household in the BDHS sample using HemoCue rapid testing methodology. For the test, a drop of capillary blood was taken from a child’s fingertip or heel and was drawn into the microcuvette which was then analyzed using the photometer that displays the hemoglobin concentration [14]. After selecting only children from *de jure* households and excluding children with missing information on hemoglobin or any of the other key predictors considered in this study, 2171 children of 6–59 months aged from the 2011 survey were retained for the final analysis. Data selection procedure is given in Fig. 1 in the form of a flow chart.

### Ethics approval

Our study was wholly based on an analysis of existing public domain health survey datasets obtained from BDHS 2011, which is freely available online with all identifier information removed. The main author communicated with MEASURE DHS and ICF International and permission was granted to download and use the data. The BDHS 2011 was reviewed and approved by the ICF Macro Institutional Review Board and the National Research Ethics Committee of the Bangladesh Medical Research Council. This survey was conducted by the National Institute of Population Research and Training (NIPORT) of the Ministry of Health and Family Welfare and implemented by Mitra and Associates, Bangladesh. The technical assistance for the survey was provided by ICF International of Calverton, Maryland, USA, as a part of its international Demographic and Health Survey program (MEASURE DHS). The U.S. Agency for International Development (USAID) provided financial support to complete the survey.

### Measurement of variables

#### Outcome variables

Anemia was considered as the outcome variable. Hemoglobin concentration is the most reliable indicator of anemia at the population level [1]. According to WHO’s criteria, 6–59 months aged children with hemoglobin level less than 11.0 g/dl are considered as anemic [1].

#### Explanatory variables

A number of health, demographic and socio economic factors are associated with children’s nutritional status. Maternal age (<20, 20–29, 30–39,≥40), parental educational (no education, primary, secondary, higher), sex of the children, age of the children (6–23 months, 24–59 months), number of living children (1, 2, 3, >3), number of household members (≤4, 5–8, ≥9), number of eligible children (1, >1), currently breastfeeding (yes, no), mother’s anemia (yes, no), mother’s Body Mass Index, BMI (<18.5 kg/m^2^, ≥18.5 kg/m^2^), size of the children at birth (small, average, large), household toilet facilities and source of drinking water (both binary variables broken down into improved and non-improved), presence of fever (yes, no) or diarrhea (yes, no) within last 2 weeks from date of interview are all considered potentially important factors in analyzing nutritional status of under five aged children and were included in the analysis. According to BDHS, a household water connection (piped), public standpipe, borehole, protected dug well or spring or rainwater collection is considered to be an ‘improved’ source of drinking water. Similarly, the ‘improved’ toilet facilities are considered to be flush toilets, ventilated improved pit latrines, traditional pit latrines with a slab, or composting toilets [14]. A Z-score cut-off point of less than −2 standard deviation (SD) is used to classify child malnutrition status such as low weight-for-age (underweight), low height-for-age (stunting) and low weight-for-height (wasting) according to WHO criteria. A wealth index was calculated using principle component analysis of asset variables and then categorized into terciles (poor, middle, rich). Place of residence (rural, urban) and geographic region based on seven divisions in Bangladesh (Barisal, Chittagong, Dhaka, Khulna, Rajshahi, Rangpur and Sylhet) were also included as covariates.

### Statistical analysis

Descriptive statistics of each of the selected variables and distribution of anemia by different factors were shown with 95 % CI by adjusting sampling weight. The BDHS 2011 sample was a two-stage stratified cluster sample; sampling weights were calculated based on sampling probabilities separately for each sampling stage and cluster. Due to the non-proportional allocation of sample to divisions and urban and rural areas, and the differences in response rates in sample, sampling weights were adjusted to ensure the representativeness of the survey results at national level. Adjustment for clustering in the sample removes underestimation of variability in the estimates by adjusting standard errors, and weighting the data adjusts for under sampling and oversampling within strata. A detailed description of the weighting procedure can be found in the BDHS report [14]. Logistic regression was applied and ORs with 95 % CI were used to evaluate the factors associated with anemia among 6–59 months aged children. Factors exhibiting a significant association with anemia (*p*-value <0.05) in univariate models were selected for developing multivariable logistic regression model. Statistical analyses were performed using the R statistical software (version 3.0.1; The R Foundation for Statistical Computing).

## Results

A total of 2171 children between the ages of 6–59 months were identified. Among the eligible children male and female ratio were 51:49. Over two thirds of the samples were aged 24–59 months (67.2 %). The prevalence of anemia among children of aged 6–59 months was 51.9 % (95 % CI 49.4-54.5). Prevalence of stunting and underweight status in the children was over a third while the prevalence of wasting was approximately 16 %. The majority of the mothers of the children were at 20–29 years of age and 12.4 % was less than 20 years. Among the selected households, more than half had 5–8 members, with 1-2 children and one 6–59 months aged child. About 98.4 % households had access to improved water sources, while only 51.0 % households had access to improved toilet facilities. The proportion of no formal education among the children’s fathers was higher than the mothers. About 44.1 % of children’s mothers were anemic and 30.2 % were malnourished (BMI <18.5 kg/m^2^) at the time of the survey. More than three-quarters (78.8 %) respondents lived in rural residences reflecting the changing demographics of Bangladesh, and 37.1 % of children lived in households of ‘poor’ economic class. Most of the selected respondents (30.7 %) lived in Dhaka division in contrast to Barisal division (5.7 %) (Table 1).

**Table 1:** Descriptive statistics of selected variables

Variables	Weighted Estimate (Mean/Proportion)	95 % CI
Anemia (*Hb < 11.0 g/dl*)		
No	48.1	45.5-50.6
Yes	51.9	49.4-54.5
*Household characteristics*		
Number of HH members		
≤4	31.5	28.7-34.2
5-8	54.2	51.4-57.1
≥9	14.3	12.0-16.7
Number of under-5 children		
One	60.9	57.8-64.0
More than one	39.1	36.0-42.2
Number of living children	2.4	2.3-2.5
1	28.5	26.1-31.0
2	32.9	30.3-35.4
3	19.5	17.3-21.8
>3	19.1	16.7-21.4
Toilet facilities		
Improved	51.0	47.7-54.4
Non-improved	49.0	45.6-52.3
Water source		
Improved	98.4	97.7-99.2
Non-improved	1.6	0.9-2.3
Wealth index		
Poor	37.1	33.9-40.4
Middle	31.8	29.0-34.5
Rich	31.1	28.0-34.2
*Parental characteristics*		
Maternal age (*years*)		
<20	12.4	10.8-14.1
20-29	62.2	59.7-64.7
30-39	22.4	20.1-24.7
≥40	3.0	2.1-3.9
Maternal education		
No education	20.3	17.9-22.7
Primary	33.2	30.3-36.1
Secondary	39.9	36.6-43.1
Higher	6.6	5.3-7.9
Father's education		
No education	29.9	27.1-32.8
Primary	30.4	27.8-32.9
Secondary	27.8	25.3-30.3
Higher	11.9	10.2-13.7
Maternal anemia		
Anemic	44.1	41.3-47.0
Not anemic	55.9	53.0-58.7
Mother’s BMI		
<18.5 kg/m^2^	30.2	27.4-33.0
≥18.5 kg/m^2^	69.8	67.0-72.6
*Child’s characteristics*		
Sex of children		
Male	51.0	48.4-53.5
Female	49.0	46.5-51.5
Age of the children (*months*)		
6-23 months	32.8	30.8-34.9
24-59 months	67.2	65.1-69.2
Size of children at birth		
Large	13.3	11.5-15.1
Average	68.5	66.2-70.9
Small	18.2	16.3-20.1
Continuous breastfeeding		
No	36.1	33.5-38.7
Yes	63.9	61.3-66.6
Diarrhea in last 2 weeks		
No	94.9	93.8-96.0
Yes	5.1	4.0-6.2
Fever in last 2 weeks		
No	61.3	58.9-63.8
Yes	38.7	36.2-41.1
Stunting		
No (HAZ ≥ −2 SD)	57.8	55.0-60.7
Yes (HAZ < −2 SD)	42.2	39.4-45.0
Under-weight		
No (WAZ ≥ −2 SD)	61.8	59.0-64.6
Yes (WAZ < −2 SD)	38.3	35.4-41.1
Wasting		
No (WHZ ≥ −2 SD)	83.6	81.8-85.5
Yes (WHZ < −2 SD)	16.4	14.5-18.2
*Community characteristics*		
Place of residence		
Urban	21.2	18.6-23.7
Rural	78.8	76.3-81.4
Division		
Barisal	5.4	3.9-7.0
Chittagong	22.2	17.4-26.9
Dhaka	30.7	25.2-36.2
Khulna	9.4	6.9-11.8
Rajshahi	12.7	9.5-16.0
Rangpur	11.5	8.6-14.4
Sylhet	8.1	5.8-10.4

WAZ (Weight for Age Z-score), HAZ (Height for Age Z-score), and WHZ (Weight for Height Z-score) was calculated with WHO Anthro and WHO Child Growth Standards

Table 2 shows prevalence of anemia by different factors. The prevalence of anemia varied significantly (*p* < 0.001) by age. For the children aged between 6 to 23 months the prevalence was 28 % greater than the children of age 24–59 months. The percentage of anemic children varied with educational status of parents. The prevalence of anemia was significantly higher for parents with no formal education compared to the higher educated parents. For stunted children the prevalence of anemia was significantly (*p* = 0.031) higher than in non-stunted children. But we did not find any significant difference in anemia prevalence among the wasted and underweight children. Children who continued breastfeeding were more anemic than the non breast fed children (58.6 % vs. 40.2 %, *p* < 0.001). The prevalence of anemia among children was significantly (*p* < 0.001) higher for anemic mothers (61.6 %) and malnourished mothers (58.0 %). There was a significant (*p* < 0.01) difference of anemia prevalence between children from households with access to an improved water source (51.1 %) and those without such access (74.3 %). But there was no significant difference in anemia prevalence by toilet facilities of households. The prevalence of anemia among children who suffered from fever in last 2 weeks was about nine percent higher than others. Not surprisingly, children from poor and middle economic class families were more anemic than children from rich families. Moreover, the prevalence of anemia was significantly higher among children in rural areas (53.1 %), compared to the urban areas (47.4 %).

**Table 2:** Prevalence of anemia by different factors

Variables	Weighted Prevalence	95 % CI	*p*-value*
*Household characteristics*			
Number of HH members			
≤4	49.5	45.0-54.1	0.408
5-8	53.3	50.2-56.4	
≥9	52.1	45.2-59.0	
Number of under-5 children			
One	51.5	48.4-54.5	0.642
More than one	52.7	48.3-57.1	
Number of living children			
1	54.8	50.1-59.5	0.078
2	47.1	42.6-51.7	
3	54.0	48.4-59.7	
>3	53.8	48.0-59.6	
Toilet facilities			
Improved	49.5	45.7-53.2	0.140
Non-improved	53.6	49.8-57.4	
Water source			
Improved	51.1	48.5-53.8	<0.01
Non-improved	74.3	60.8-87.8	
Wealth index			
Poor	57.9	53.8-62.0	<0.001
Middle	52.4	47.9-56.9	
Rich	44.3	40.0-48.7	
*Parental characteristics*			
Maternal age (*years*)			
<20	65.6	59.0-72.3	<0.001
20-29	50.2	46.9-53.6	
30-39	48.8	43.6-53.9	
≥40	54.2	40.3-68.1	
Maternal education			
No education	51.4	45.8-56.9	0.012
Primary	53.8	49.5-58.0	
Secondary	53.2	49.2-57.2	
Higher	37.0	28.1-45.9	
Father's education			
No education	51.3	46.5-56.1	<0.01
Primary	56.2	51.6- 60.8	
Secondary	52.3	47.8-56.8	
Higher	41.6	34.8-48.4	
Maternal anemia			
Anemic	61.6	58.2-65.0	<0.001
Not anemic	44.3	40.9-47.6	
Mother’s BMI			
<18.5 kg/m^2^	58.0	53.2-62.8	<0.01
≥18.5 kg/m^2^	49.2	46.3-52.1	
*Child’s characteristics*			
Sex of children			
Male	53.0	49.6-56.5	0.363
Female	50.8	47.2-54.4	
Age of the children (*months*)			
6-23 months	70.8	66.8-74.8	<0.001
24-59 months	42.7	39.6-45.8	
Size of children at birth			
Large	55.5	49.1-62.0	0.447
Average	51.0	47.84-54.2	
Small	52.8	47.1-58.5	
Continuous breastfeeding			
No	40.2	36.2-44.1	<0.001
Yes	58.6	55.4-61.8	
Diarrhea in last 2 weeks			
No	51.9	49.3-54.5	0.815
Yes	53.3	41.7-64.8	
Fever in last 2 weeks			
No	48.8	45.6-52.1	<0.01
Yes	56.9	53.0-60.7	
Stunting			
No (HAZ ≥ −2 SD)	49.2	46.1-52.3	0.031
Yes (HAZ < −2 SD)	55.0	50.7-59.3	
Under-weight			
No (WAZ ≥ −2 SD)	51.5	48.4-54.7	0.905
Yes (WAZ < −2 SD)	51.8	47.7-55.9	
Wasting			
No (WHZ ≥ −2 SD)	51.8	49.0-54.6	0.822
Yes (WHZ < −2 SD)	51.1	45.2-56.9	
*Community characteristics*			
Place of residence			
Urban	47.4	42.7-52.2	0.046
Rural	53.1	50.1-56.2	

*p-value obtained from chi-square test of contingency table

**Fig. 2:**
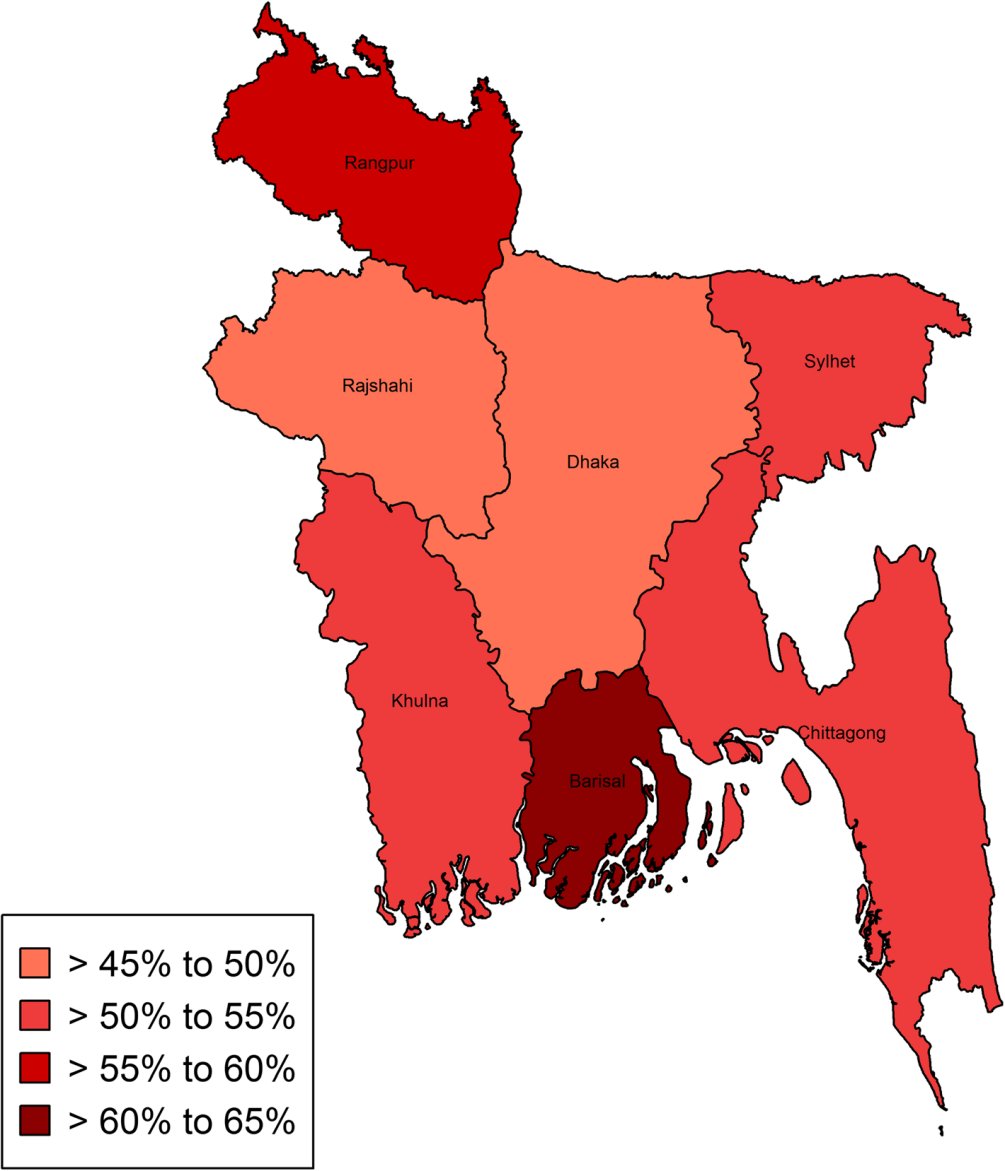
Division wise prevalence of anemia

Figure 2 shows that the administrative division-wise prevalence of anemia. Barisal, a division from the southern region of the country had the highest prevalence of anemia among children under the age of 5. A northern region Rangpur, also showed similar pattern. The results indicated that about 6 out of 10 children in the Barisal and Rangpur division were anemic; with prevalence estimates for these two regions being 60.4 % and 58.9 % respectively. The lowest prevalence was recorded in Dhaka division, 47.8 %, while for Chittagong, Rajshshi, Khulna and Sylhet rates of anemia were above 50 %. Relative to the WHO cut-off 40 %, all divisions showed concerning levels of anemia.

Table 3 shows the odds ratios from simple and multivariable logistic regression analysis for assessing associations between different factors and anemia among children aged 6–59 months in Bangladesh. Age of the children is recognized as an important factor for childhood anemia, and results showed that children with age between 6–23 months were more at risk of suffering from anemia than 23–59 months aged children, OR 3.01 (95 % CI: 2.38-3.81, *p* < 0.001). Adjusting for other factors, anemic mother’s children were 80 % more likely to be anemic compared to children of non-anemic mothers. Similarly, children from undernourished mothers’ were 43 % more likely to be anemic than others. Moreover, parental education was associated with lower rates of PreSAC anemia: children of parents with no formal education were at high risk of anemia compared to the children of educated parents. Stunting or chronic malnutrition also displayed significant associations with anemia among the PreSAC population. Children who suffered from chronic malnutrition were more likely to be anemic, OR 1.38 (95 % CI: 1.13-1.69, *p* < 0.01). Children who suffered from fever in the 2 weeks prior to measurement were 28 % more likely to manifest anemia. Source of drinking water and toilet facilities of the household are thought to be strong household level predictors of childhood anemia. Children from households without access to ‘improved’ water sources and toilet facilities were 1.34 and 2.48 times more likely than others to be anemic. Compared to children of poor households according to the composite wealth index, middle and rich household’s children were 26 % and 34 % less likely to be anemic. The likelihood of being anemic was 1.21 times higher for rural children than the urban children. Children from Dhaka and Sylhet divisions were less likely to be anemic than the children from Barisal division, which had the highest anemia prevalence among all the divisions.

**Table 3:** Factors associated with anemia among 6–59 months aged children in Bangladesh

	Simple Logistic Regression			Multivariable Logistic Regression		
Variables	OR	95 % CI	*P*-value	OR	95 % CI	*P*-value
*Household characteristics*						
Number of HH members						
≤4	1			-	-	-
5-8	1.05	0.87-1.28	0.582	-	-	-
≥9	0.99	0.76-1.28	0.923	-	-	-
Number of under-5 children						
One	1			-	-	-
More than one	1.01	0.85-1.20	0.907	-	-	-
Number of living children						
1	1					
2	0.80	0.65-0.99	0.043	1.01	0.78-1.31	0.929
3	0.89	0.69-1.14	0.351	1.13	0.81-1.56	0.473
>3	0.92	0.72-1.18	0.499	1.19	0.81-1.74	0.381
Toilet facilities						
Improved	1			1		
Non-improved	1.34	1.13-1.59	<0.001	1.03	0.83-1.27	0.811
Water source						
Improved	1			1		
Non-improved	2.30	1.25-4.44	<0.01	2.48	1.28-5.02	<0.01
Wealth index						
Poor	1			1		
Middle	0.76	0.61-0.93	<0.01	0.74	0.57-0.95	0.018
Rich	0.56	0.45-0.69	<0.001	0.66	0.48-0.92	0.013
*Parental characteristics*						
Maternal age (*years*)						
<20	1			1		
20-29	0.50	0.38-0.66	<0.01	0.76	0.54-1.07	0.122
30-39	0.46	0.34-0.63	<0.01	0.71	0.46-1.10	0.123
≥40	0.52	0.30-0.90	0.019	0.68	0.34-1.38	0.286
Maternal education						
No education	1.78	1.22-2.61	<0.01	1.23	0.71-2.14	0.468
Primary	1.86	1.30-2.67	<0.01	1.15	0.69-1.92	0.589
Secondary	1.72	1.21-2.46	<0.01	1.10	0.70-1.75	0.670
Higher	1			1		
Father's education						
No education	1.45	1.09-1.94	0.010	1.02	0.66-1.56	0.943
Primary	1.73	1.30-2.30	<0.001	1.25	0.84-1.85	0.276
Secondary	1.47	1.10-1.96	<0.01	1.24	0.86-1.80	0.252
Higher	1			1		
Maternal anemia						
Anemic	1.93	1.62-2.29	<0.001	1.80	1.49-2.18	<0.001
Not anemic	1			1		
Mother’s BMI						
<18.5 kg/m^2^	1.43	1.19-1.72	<0.001	1.07	0.86-1.33	0.554
≥18.5 kg/m^2^	1			1		
*Child’s characteristics*						
Sex of children						
Male	1			-	-	-
Female	0.92	0.78-1.09	0.320	-	-	-
Age of the children (*months*)						
6-23 months	3.31	2.73-4.02	<0.001	3.01	2.38-3.81	<0.001
24-59 months	1			1		
Size of children at birth						
Large	1			-	-	-
Average	0.89	0.69-1.13	0.341	-	-	-
Small	0.89	0.66-1.20	0.448	-	-	-
Continuous breastfeeding						
No	1			1		
Yes	2.13	1.78-2.55	<0.001	1.18	0.95-1.47	0.143
Diarrhea in last 2 weeks						
No	1			-	-	-
Yes	1.20	0.83-1.75	0.328	-	-	-
Fever in last 2 weeks						
No	1			1		
Yes	1.28	1.07-1.52	<0.01	1.13	0.93-1.37	0.229
Stunting						
No (HAZ ≥ −2 SD)	1			1		
Yes (HAZ < −2 SD)	1.41	1.19-1.68	<0.001	1.38	1.13-1.69	<0.01
Under-weight						
No (WAZ ≥ −2 SD)	1			-	-	-
Yes (WAZ < −2 SD)	1.10	0.92-1.31	0.306	-	-	-
Wasting						
No (WHZ ≥ −2 SD)	1			-	-	-
Yes (WHZ < −2 SD)	0.96	0.76-1.21	0.723	-	-	-
*Community characteristics*						
Place of residence						
Urban	1			1		
Rural	1.21	1.01-1.46	0.039	0.93	0.74-1.17	0.517
Division						
Barisal	1			1		
Chittagong	0.77	0.56-1.07	0.120	0.72	0.50-1.05	0.087
Dhaka	0.64	0.46-0.89	<0.01	0.63	0.43-0.92	0.016
Khulna	0.83	0.58-1.19	0.309	0.97	0.64-1.45	0.865
Rajshahi	0.68	0.48-0.97	0.035	0.74	0.49-1.10	0.135
Rangpur	0.97	0.69-1.38	0.878	0.94	0.64-1.40	0.769
Sylhet	0.66	0.47-0.91	0.012	0.61	0.41-0.89	0.011

## Discussion

Childhood anemia is a major public health challenge in Bangladesh. Our results reveal that about 52 % of the children aged 6–59 months nationally are anemic, which is consistent with previously reported national prevalence of anemia (51 %) [14]. The findings confirm previous Bangladeshi studies showing high prevalence of anemia among the under-5 children [15], although the current study shows higher levels than, for example, the National Micronutrient Survey [13]. The study confirmed prevalence estimates differed by a number of key variables associated with anemia, as well as regional variation. Our analysis demonstrates that the age of child, chronic malnutrition status of child, mother’s anemia status, source of water of household, wealth index have statistically significant associations with childhood anemia.

This study reveals that, the prevalence of anemia amongst every young (those under 2 in the PreSAC sample) was higher than in the overall population. This would likely be due to the high prevalence of maternal micronutrient deficits [16] as well as low concentrations of iron in breast milk, insufficient to meet daily requirements of iron for the children [8]. The likelihood of anemia is significantly higher among children less than 2 years old compared to those aged 2–5 years. These findings are consistent with previously reported results [9, 17–20].

Household’s source of drinking water showed an association with anemia in the PreSAC sample although the percentage of households which had no access to improved water source was only 1.58 %. But among those households, about 74 % of children presented with anemia. These elevated levels could be associated with higher rates of infectious diseases, although presence of fever was controlled in this study. The study’s findings in relation to quality of water supply were consistent with previous research [21, 22]. Younger children and those with fever in the previous 2 weeks were also more likely to be anemic. According to [23], fever is common symptom of acute and chronic diseases which have been associated with lower hemoglobin levels as well as anemia.

The prevalence of anemia among children of low height for age (stunted) was high. Stunting, as an indicator of chronic malnutrition, is positively associated with childhood anemia [21, 22, 24, 25], and this association was found in the current study. Nutritional inadequacies may also impair immunity which in turn can have associations with low concentrations of hemoglobin (anemia).

Maternal anemia was highly associated with the occurrence of childhood anemia and the prevalence of anemia among the children of anemic mothers was almost 62 %, again corroborating several previous findings [26–28]. The underlying reasons may be mothers and children share common home environment, socioeconomic, and dietary conditions, and maternal/child anemia may reflect the common nutritional status of the household. Moreover, maternal iron deficiency is associated with low birth weight; even children born with adequate weight have reduced iron reserves when their mothers are anemic [29, 30].

Children of the rich and middle class households had lower prevalence of anemia compared to the poor households, plausibly reflecting improved household nutritional status [31]. This finding is also consistent with previous studies [32, 33].

The study also revealed that children of non-educated, primary and secondary educated parents were more likely to be anemic than children of parents with higher education. Level of education is confounded with socioeconomic status in general, but may also reflect in relatively poorer understanding of optimum child care and nutritional practices. Again, the study results in this regard are consistent with literature [1]. This study also shows an association between maternal age and PreSAC anemia, with older mothers less likely to have anemic children. This is plausibly due to a number of factors, with timing of childbearing being associated with socioeconomic status and household wealth [34].

Higher prevalence of anemia was found in rural regions of Bangladesh. This can also be linked to malnutrition due to limited availability of nutritious foods due to lower socioeconomic status, and lack of access to hygienic sanitation facilities [35], associated with elevated rates of disease which in turn is associated with increased risk of anemia. The high prevalence of anemia in rural sectors especially in the southern division Barisal and northern division Rangpur can be linked to the fact that most of the areas in these regions are rural. Demographic changes in Bangladesh are seeing the increased concentration of industries associated with economic growth and thus higher socioeconomic status into the major population centres, Dhaka and Chittagong. Although all of the divisions sampled were at high risk of anemia, the low prevalence observed in Dhaka division, could be due to the high proportion of urban residents in the Dhaka region.

### Limitations

Detail on infant and PreSAC feeding was not available for our sample, which limits the insight that can be drawn from the data. Moreover, the BDHS survey does not distinguish between slum areas from the other residential areas. Slum areas will be automatically included in sample if they exist. So, these data are not representative for the slum population of urban areas, who are probably more likely to suffer from anemia. While the data are designed to be nationally representative, it is possible that there is some degree of bias in the sampling that might see slum residents underrepresented in the sample. However, we cannot prove it to be a potential source of bias since there is no identifier of slum area in the data. If we had such an identifier, we could compare the estimates by post-stratification. Despite these limitations we believe that our findings help illuminate the association between socioeconomic, demographic, and health variables with anemia in a large PreSAC population in the developing world.

## Conclusions

In summary, our analysis highlights concerning continuing public health challenge presented by anemia in a PreSAC population in Bangladesh. This study explores the factors associated with anemia. This study supports the value of population-based interventions such as micronutrient supplementation, food fortification and nutrition education to improve the situation that should be instituted. The findings of the study will assist the government of Bangladesh and policy makers to take necessary steps and design proper interventions that target children aged under-5, and their parents. However, further study is needed to understand the specific set of determinants of anemia among children in Bangladesh.
